# The Scientific Evolution of Periacetabular Osteotomy: A Global Review

**DOI:** 10.3390/jcm11206099

**Published:** 2022-10-17

**Authors:** Sufian S. Ahmad, Marco Haertlé, Christian Konrads, Alexander Derksen, Henning Windhagen, Nils Wirries

**Affiliations:** 1 Department of Orthopaedic Surgery, Hannover Medical School, 30625 Hannover, Germany; 2 Department of Orthopaedic Surgery, University of Tübingen, 72076 Tübingen, Germany

**Keywords:** borderline hip dysplasia, dysplasia, hip arthroscopy, hip surgery, hip preservation, PAO

## Abstract

It is well-known that hip disorders are frequently of bony origin related to an underlying pathomorphology. A fundamental understanding of morphology and biomechanics is therefore of essential importance for a targeted approach in defining treatment plans. Treatment is frequently based on altering bony morphology, for which a set of effective techniques have been proposed. Periacetabular osteotomy (PAO) allows for reorientation of the acetabulum and powerful correction of acetabular coverage. The revolutionary aspect of PAO compared to prior osteotomies lies in maintenance of the integrity of the posterior column. This allows for a substantial increase in primary stability, a larger bony surface for healing, and simple reorientation of the acetabular fragment that is free of posterior ligamentous restraints. The results for dysplasia are very promising. Indications have been refined by studies revealing that the presence of degenerative changes and age > 40 years at the time of surgery represent prognostic factors of poorer outcome. Indications have also been broadened to include acetabular retroversion (with posterolateral dysplasia) and borderline hip dysplasia. A glimpse at the future would reflect major advances related to individual planning, surgical training, and precise surgical conduction. In the era of digitalization, augmented reality may assist in performing bony cuts and act as an aid for some of the blind ischial and retro-acetabular cuts. Innovations in perioperative management will enhance recovery after the procedure and allow for early recovery programs with optimized protocols of pain management. Considering that the success of PAO in the young is comparable to the success of hip arthroplasty in the old, PAO should be considered one of the pillars of modern orthopedic surgery.

## 1. Introduction

**Figure 1:**
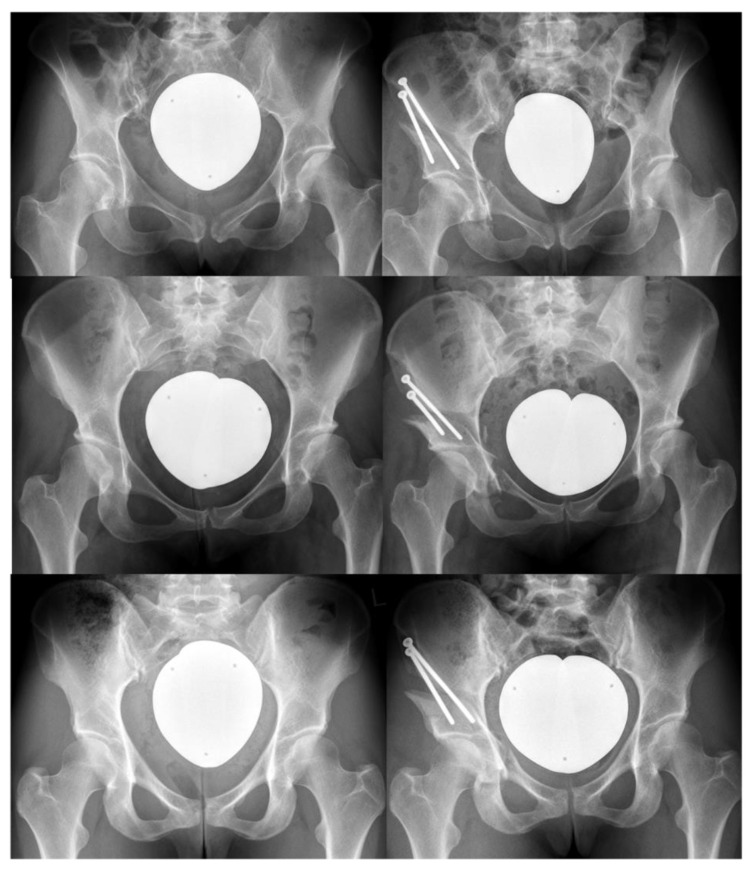
Three cases of periacetabular osteotomy. The preoperative X-ray is depicted on the left of each row, and the corresponding postoperative on the right. Note that due to the inherent stability of the construct, the first author of this review (SSA) generally uses only two 4.5 mm screws for fixation.

Periacetabular Osteotomy (PAO) is a powerful and well-established surgical procedure for the treatment of malorientation of the acetabulum [1,2]. Since its first description by Reinhold Ganz in 1988, a revolutionary change in the world of hip preserving surgery was observed [2,3]. The advantages of PAO over prior acetabular osteotomies were primarily underlined by preserving the integrity of the posterior column of the acetabulum, allowing for more primary stability. Figure 1 shows examples of a PAO procedure from the authors’ institution.

Reinhold Ganz also advocated the idea that PAO was also less disruptive of the acetabular blood supply, given that it is performed from the inside of the pelvis [4].

Preserving the integrity of the posterior column was considered to free the mobile acetabular fragment from ligamentous restraints that are attached to the posterior column, thereby easing reorientation.

The ultimate benefits of the procedure have allowed for it to be adopted by many surgeons around the globe, resulting in more than a thousand subsequent articles being published [1,5].

The aim of this review was to present some key scientific developments around the concept of the PAO procedure that have emerged since its first description in 1988.

### Early Description of Periacetabular Osteotomy

In 1984, the first periacetabular osteotomy (PAO) procedure was performed by Reinhold Ganz at the Inselspital of the University of Bern in Switzerland. The early outcomes of the first series of patients treated with this procedure between 1984 and 1987 were published in “Clinical Orthopedics and Related Research” in 1988 [2]. With over 1300 citations, this article is considered one of the most cited articles in the field of hip orthopedic research and has therefore significantly influenced clinical practice by introducing a valuable osteotomy concept for the treatment of residual dysplasia of the hip [6]. The experience that was reported in the article also highlighted a few technical challenges associated with the procedure. These included significant problems associated with long operating times of up to 5 ½ h, 3000 cc average blood loss, and intra-articular propagation of osteotomies. However, after a learning curve of 18 cases, the required surgical time decreased (mean 2 ½ h) and so did the average blood loss (800 cc). It was therefore noted by the authors of the first article on PAO that the procedure was technically demanding and requires extensive three-dimensional understanding of the morphology of the pelvis as well as extensive practice on models and cadavers. However, no major neurovascular injury was reported. Ten years later, Klaus Siebenrock published a subsequent follow-up of the Ganz series [3]. It was noted that despite the complications that were encountered in the first 18 cases, 82% of hips were preserved at 11 years and 73% demonstrated good-to-excellent results [3]. This study underlined several factors that were associated with poorer results including age at the time of surgery and the presence of degenerative changes [3]. It was even mentioned at that time that insufficient anterior coverage after correction yields poorer results [3]. Therefore, it must be underlined that the Ganz series provided early and comprehensive clinical knowledge about the concept of PAO that is well-known today.

## 2. Indications for Periacetabular Osteotomy

### 2.1. Classical Indications

**Figure 2:**
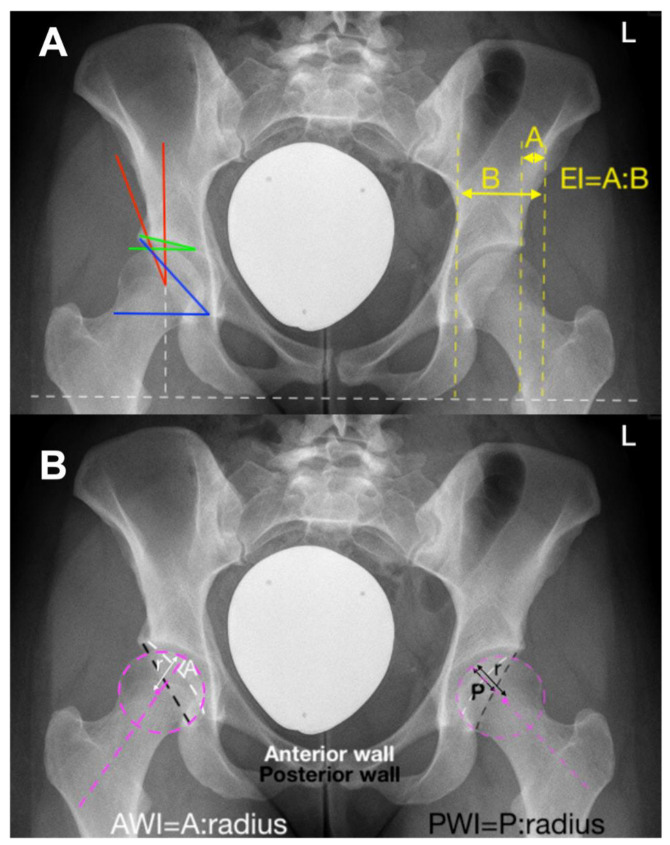
A 24-year-old female with a symptomatic DDH on the right side. Radiological criteria: (**A**) Lateral center edge angle (LCEA) (red): 18.9°, Acetabular index (green): 12.7°, Sharp angle (blue): 47.5°; Extrusion index (EI) (yellow): 22.6%. (**B**) Anterior wall coverage (AWI): 16.2%, Posterior wall coverage (PWI): 50.2%.

Initially, PAO was established for the treatment of hip dysplasia (Figure 2).

**Figure 3:**
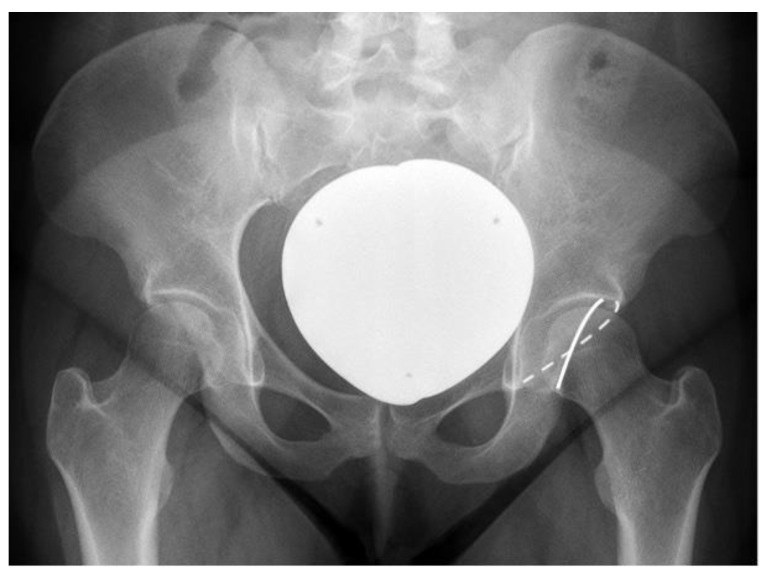
A 23-year-old female with a symptomatic acetabular retroversion on the right side. Radiological criteria: Lateral center edge angle (LCEA): 25.1°, Acetabular index: 12.1°, Extrusion index: 22.7%, Anterior wall coverage: 31.6%, Posterior wall coverage: 29.2%, Sharp angle: 39.7°. It is to be underlined that the problem is not a potential anterior conflict only, but posterolateral dysplasia. Dashed white line: Anterior wall; White line: Posterior wall.

However, the indication was later broadened to include acetabular retroversion and anterior over-coverage (Figure 3) [7,8]. This will be dealt with later in this review.

The main imaging modality for the diagnosis of hip dysplasia is the plain ap pelvic radiograph. This represents the best-validated imaging form for the determination of acetabular coverage [9]. It provides a depiction of lateral, anterior, and posterior coverage of the femoral head [9]. Magnetic resonance imaging (MRI) may be considered as an adjunct to evaluate the overall status of the cartilage and labrum. However, MRI does not provide information regarding bony coverage and is not the modality that allows for diagnosis.

Given that dysplasia is a three-dimensional problem of under-coverage, it is difficult to set radiographic margins to clearly define surgical indication. In a study by Moritz Tannast looking into the radiographic features of dysplastic hips, the radiographic morphometric values of patients with a dysplastic hip undergoing periacetabular osteotomy were analyzed. These values represent reference values from distribution curves in a group of patients with hips that were agreed upon as dysplastic and had undergone a PAO in the institution where the procedure was first described. The morphometric measures based on an ap pelvic X-ray include [9]:

− Lateral center edge angle (LCEA): 22°
− Acetabular index: >14°
− Extrusion index: >27%
− Anterior wall coverage: <14%
− Posterior wall coverage: <35%
− Sharp angle: >43°

Surgical indications are set on an individual basis. Radiographic features need appreciation as mentioned above. Understanding factors associated with failure of PAO are important to guiding the surgical decision [10]. This will be dealt with later in this review. It is obvious that there are standard deviations for every radiographic determinant of dysplasia. Cutoff values should only guide clinicians. The potential success of a PAO in cases of borderline hip dysplasia renders radiographic margins alone insufficient [11].

However, there are some specific indications that are unique to the PAO procedure including the closure of triradiate cartilage. This is due to the retro-acetabular cut that would otherwise cross the triradiate cartilage. The PAO is therefore not a pediatric pelvic osteotomy due to the fact that it crosses the triradiate cartilage, but can be applied in young patients once the triradiate cartilage is closed [12].

True acetabular retroversion is an acknowledged cause of an anterior conflict and anterior impingement between the acetabular rim and subspine region with the femur [13]. The three radiographic signs are compulsory for this diagnosis, namely, the ischial spine sign, the crossing over sign, and the posterior wall sign [13]. In patients with true acetabular retroversion, with the presence of the three mentioned radiographic measures, anteverting PAO is more effective in achieving long-term outcomes compared to anterior wall trimming [7].

Appreciating the femur is also important prior to surgical planning. Instability may be exacerbated by the morphology of coxa valga. Furthermore, additional conflicts are influences by femoral antetorsion. A torsional MRI or CT scan may therefore be necessary to determine abnormalities of the femur in the overall workup.

### 2.2. Extended Indications

Although anterolateral under-coverage represents the best-known indication for PAO, more indications have been shown to pose at least similar benefits as undergoing this procedure (Figure 3) [8,14,15]. Focal anterior over-coverage is acknowledged as the second established indication for PAO [16]. This pathomorphology is depicted by retroversion of the hemipelvis, resulting in anterior over-coverage and posterolateral under-coverage. The results of anteverting PAO are excellent and surpass those of anterior rim trimming in femoroacetabular impingement [7]. At this juncture, it is important to underline that although anterior rim trimming and anteverting PAO demonstrate similar results 5 years postoperatively, the survival curves diverge apart significantly in favor of the anteverting PAO after the 5-year mark. This is an underestimated fact in the field of hip preserving surgery [7]. Focal pincer over-coverage is to be corrected and not resected. The privilege of the anterior Smith–Petersen approach allowing excellent exposure of the capsule opens the door to work on CAM lesions or concomitant asphericity of the femoral head–neck junction. Therefore, acetabular retroversion may and should not only be seen as an extended indication but a major indication for PAO.

When it comes to what is recognized as borderline hip dysplasia, PAO has also been considered an appropriate treatment [11,17,18]. It is important to refer to the definition of borderline hip dysplasia. In our orthopedic world, the definition of borderline hip dysplasia has been set based solely on lateral acetabular coverage. One may argue whether the definition is correct, given the three-dimensional morphology of the acetabulum. Nevertheless, the most agreed-upon definition is an LCE of 20°–25° [19]. The reality is that we may be dealing with hips lacking anterosuperior or posterosuperior coverage, ultimately explaining the reason for the marked improvement in outcome after re-orientation. There is still a limited number of studies reporting the outcome of PAO in these hips [20]. However, the studies that are published demonstrate the success of the procedure. This is surely an area that will need much more work in the future.

## 3. Surgical Technique

PAO is defined by aspects of technicality. Therefore, the technique will be described in this section of the review.

**Figure 4:**
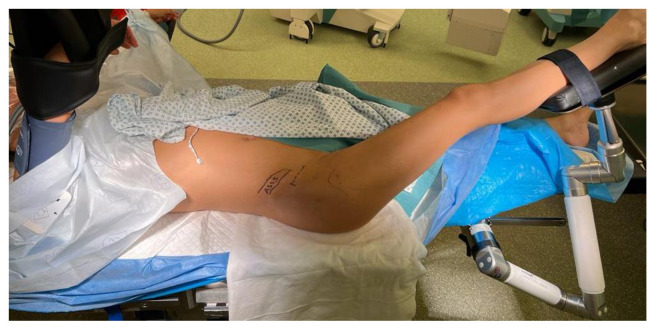
The skin incision (bikini incision; right hip) as shown by the dashed line runs parallel to the pelvic rim centered approximately 2 cm below the ASIS with a length of 5–8 cm in the iliac crease. The authors of this review use a robotic arm to allow for repositioning of the leg during surgery.

The approach to the acetabulum is an anterior Smith–Petersen approach. Therefore, the skin incision is centered distally and laterally to the anterior superior iliac spine (ASIS), proximally extending lateral to the iliac crest and distally extending laterally towards the fibula head. Nowadays, it is much more common to use a modified bikini-like incision aiming towards the inguinal crease that is much more aesthetic, as shown in Figure 4 at the right hip.

**Figure 5:**
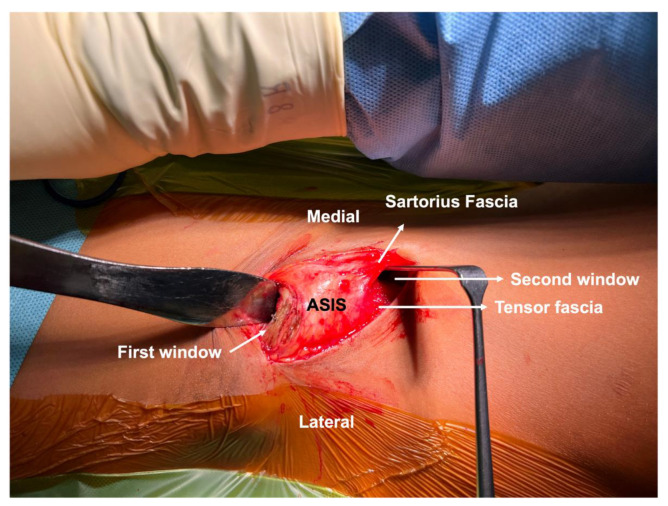
An intraoperative image from the surgical field depicting the Smith–Petersen approach with the two windows. The first window is developed proximally with subperiosteal insertion of a retractor beneath the iliacus muscle. The second window is developed between the Sartorius muscle and the tensor fascia lata. The two windows may be connected by mobilizing the soft tissue band from the anterior superior iliac spine (ASIS) containing the Sartorius muscle and inguinal ligament.

The fascia of the tensor muscle is incised over the muscle belly and the muscle is retracted laterally, developing a window between the tensor and sartorius muscles. This window represents the distal limb of the approach. Proximally, the abdominal muscles are sharply detached from the iliac crest and the iliacus muscle is mobilized from the inner table of the ilium by means of subperiosteal dissection, thereby developing the first window (Figure 5). To gain medial exposure, the inguinal ligament and Sartorius insertions on the ASIS are sharply released, maintaining integrity of the insertion of the tensor muscle on the lateral portion of the ASIS. The initial description by Ganz involved osteotomy of the ASIS but was modified later by Siebenrock who almost always sharply released the structures from the bone, as he stated in a live surgery during the Bernese Hip Symposium. Some authors never detach the inguinal ligament and Sartorius muscles, working more from the distal window for the pubic cut. The advantage is the reduced invasiveness—at the cost of reduced exposure and increased traction of the N. cutaneous femoris lateralis [21].

Medial traction of the soft tissues and iliacus muscle allows for blunt development of the interval between the rectus muscle and the iliacus muscle. Early descriptions of the procedure involved detachment of the rectus insertion. However, this is only necessary if an arthrotomy is to be performed.

**Figure 6:**
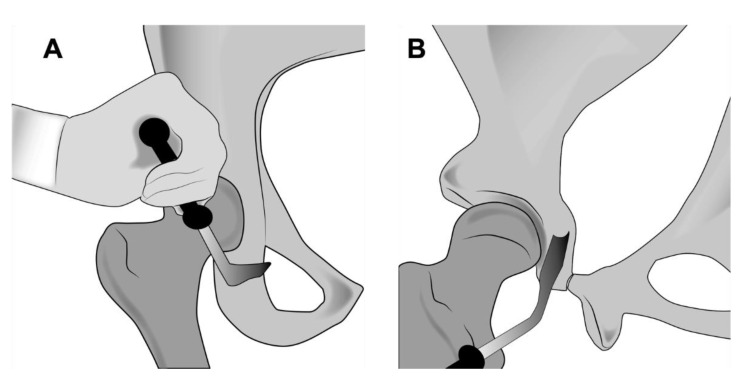
(**A**) Illustration showing the subcotyloid cut performed with an angled Ganz osteotome. (**B**) An illustration of a side view of the posterior column depicting the incomplete ischial cut erring towards the ischial spine. Several medial and lateral blows are necessary to cut the entire width of the ischium. It is important to extend the leg during the lateral cut to protect the sciatic nerve.

Bluntly, scissors are passed along this interval into the infracapsular space and spread to develop a large enough space for introduction of an angled or curved osteotome. Flexion of the leg would relax the soft tissues and ease introduction of the osteotome. This osteotome is scratched on the ischium in the infracotyloid notch to ensure correct placement. The leg must be in extension for this osteotomy to prevent injury to the sciatic nerve. The osteotome is then directed towards the ischial spine and hammered 2 to 3 cm into the bone. Fluoroscopy may be used for assistance (Figure 6). Two to three cuts from medial to lateral may be necessary to cut the entire width of the ischium. The posterior column should remain intact. Perforation of the posterior column puts the sciatic nerve at maximum risk.

**Figure 7:**
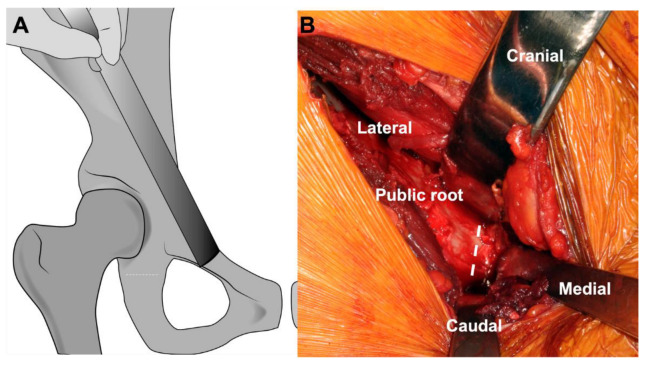
(**A**) Illustration showing the site of pubic osteotomy a few millimeters medial to the pubic root erring medially. (**B**) Intraoperative image showing the subperiosteal exposure of the pubic bone. The medial retractor is spiked into the pubic bone. The cranial and caudal retractors are subperiosteally inserted into the obturator foramen to protect the obturator neurovascular bundle. The dashed line shows the site of the osteotomy.

Then, periosteal incision close to the pelvic brim is performed und subperiosteal elevation is performed deep onto the quadrilateral plate and towards the obturator foramen. A retractor is introduced into the obturator foramen from the inside of the pelvis. A second is brought beneath the iliopsoas muscle onto the pubic bone, medial to the iliopectineal eminence, and hammered into the pubic bone to secure its position and to protect the medial structures. The pectineus origin on the pubic bone is incised and an additional retractor is inserted into the outer side of the obturator foramen to protect the neurovascular structures. Pubic osteotomy is then safely performed (Figure 7).

**Figure 8:**
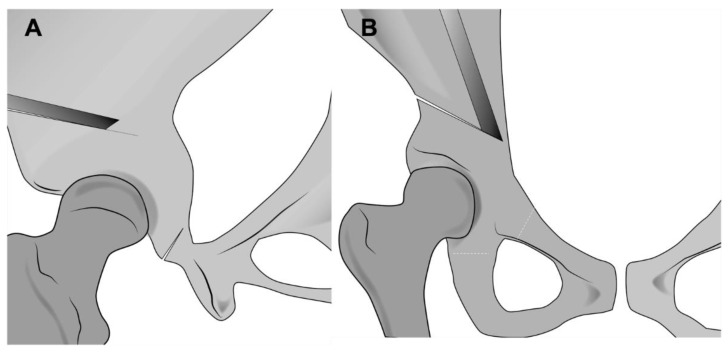
(**A**) Illustration depicting supraacetabular osteotomy that is directed towards the greater sciatic notch, ending 1 cm before the pelvic brim. (**B**) Retrotrochanteric osteotomy is commenced by angulating an osteotome towards the ischium, cutting through the thick bone of the pelvic brim into the posterior column.

Then, supraacetabular osteotomy is performed. Starting from the lower portion of the ASIS, the osteotomy should aim towards the floor, ending 1 cm before the pelvic brim. Depending on the morphology of the pelvis, this osteotomy may have a slightly cranial or distal direction to ensure sufficient distance from the iliosacral joint and sufficient proximal bone on the mobile acetabular fragment. Then, a straight osteotome is used to perform retrotrochanteric osteotomy about 110° to the supraacetabular cut aiming down towards the Ischium (Figure 8). An iliac oblique fluoroscopy view may be very helpful to verify the direction of the osteotome.

**Figure 9:**
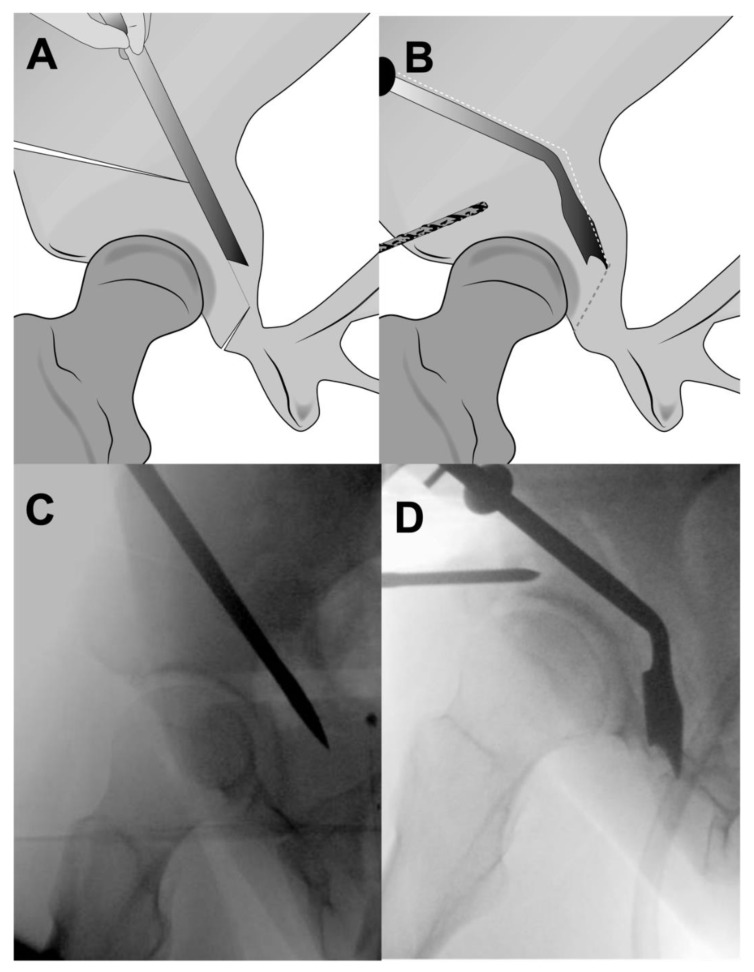
(**A**) Illustration showing the retrotrochanteric cut through the posterior column directed towards the ischium. (**B**) An angled osteotome helps to free the fragment by separating the final bony bridges. (**C**) Fluoroscopic image depicting the iliac oblique view of retrotroachteric osteotomy. (**D**) Fluoroscopic image depicting the process of freeing up the acetabular fragment by separating the residual bridges between ischial osteotomy and retrotrochanteric osteotomy.

Once the retrotrochanteric cut meets the ischial cut, the mobile fragment may be levered, and the bone begins to crack. Two areas are frequently troublesome, including the superior junction between the supra-acetabular and retro-acetabular osteotomies, where the ilium is thickest. The second frequent area that might be insufficiently cut is the lateral cortex of the ischium. The fragment may be mobile, and the false impression of a complete osteotomy may lead to poor correction. The ischial hinge would result in difficulty correcting the version, as well as difficulty rotating the fragment around the femoral head, leading to lateralization and leg lengthening. Completing the ischial cut from the inside of the pelvis and rotating the fragment with a Steinman pin inserted in the supra-acetabular bone is helpful in solving this rather common problem (Figure 9).

Freeing the acetabulum is possible by disrupting the last bony bridges between retro-acetabular osteotomy and ischial osteotomy.

The fragment is fixed temporarily with three K-wires. The orientation of the acetabulum is checked using either an intra-operative pelvic X-ray, if available, or fluoroscopy. Once the desired correction is achieved, the K-wires are replaced by three 4.5 mm cortical steel screws. The ilioinguinal ligament and sartorius muscle are reattached to the iliac crest. The abdominal aponeurosis is also reattached.

## 4. Outcome Research

### 4.1. Dysplasia

Several surgeons around the globe adopted the PAO procedure and subsequently reported their experience and results. This proved reproducibility of this method of surgery. Several studies provided Kaplan–Meier survival analysis of the dysplasic hip after PAO with total hip arthroplasty as an endpoint. These studies were published from nine institutions and represented substance for a recent meta-analysis [1,22,23,24,25,26]. In this meta-analysis, the pooled estimates of dysplastic hips of patients who were less than 40 years of age, with an LCE of <25° and limited degenerative changes were calculated. For these patients, the 5-year survivorship is expected to be 96%, the 10-year survivorship 91%, 15-year survivorship 85%, and 20-year survivorship 68% [1]. These numbers are based on the outcomes of cohorts that had undergone surgery in the early times, meaning that the outcome of a procedure performed with today’s knowledge may surpass published outcomes for dysplasia.

### 4.2. Acetabular Retroversion

In a retroverted acetabulum, the problem of an anterior conflict is commonly responsible for FAI-type symptoms. The frequently associated posterolateral insufficiency creates the additional problem of dysplasia. The combined problem of anterior FAI and posterolateral dysplasia renders the pathomorphology of acetabular retroversion disabling the patient’s hip. PAO is the treatment of choice, given that rim-trimming was shown to have poorer outcomes [7,27].

### 4.3. Factors Influencing PAO Success

To be able to achieve the best possible results, it is important to narrow indications and apply scientific knowledge in patient selection. Multivariate analyses provide effective methodological tools to determine prognostic factors influencing the outcome of a procedure. These are helpful in decision making in the clinical setting. Preoperative factors that have been reported as determinants of outcome include age, joint-space narrowing, joint incongruency, a higher Tönnis osteoarthritic grade, the presence of preoperative limp, and poor preoperative hip scores [28,29]. Surgical factors that have been reported to influence outcome include acetabular re-orientation resulting in excessive anterior over-coverage with an acetabular wall index >27% and radiographic signs of retroversion [29,30], and a postoperative LCE angle of <30° or >40° [31]. Although much focus was around avoiding retroversion of the acetabulum, it is currently being emphasized that insufficient anterior coverage due to excessive anteversion of the acetabular fragment is a major predictor of failure. This is being backed by evidence from well-designed studies with good follow-up [32]. It can therefore be highlighted that insufficient anterior coverage has the highest prognostic risk of conversion to hip arthroplasty compared to the factors. This emphasizes the importance of not only improving lateral coverage but also anterior coverage in dysplastic hips.

## 5. Potential Complications of the PAO Procedure

PAO is considered a demanding and rather complex pelvic operation with potential complications that have been reported [33]. Although the literature lacks the report of a mortality case in association with PAO, the complications of this procedure may be serious and must always be appreciated [34].

The most frequently reported complication is neuropraxia of the lateral femoral cutaneous nerve, resulting in hyposensitivity on the lateral side of the thigh [35]. This is a complication that is directly related to the anterior Smith–Petersen approach and is a common postoperative issue that occurs in 90% of patients but rarely represents a major complaint in the mid-term, despite possible residual symptoms. Further complications include sciatic nerve palsy, which is associated with cuts that are in close proximity to the ischium, namely the subcotyloid and retro-acetabular osteotomies. The femoral nerve is at risk during pubic cut, due to traction or direct injury. Intra-articular fractures, and extension of the osteotomy into the joint or into the posterior column, have also been described. Venous thrombosis and thromboembolic events can happen. Non-union was also a reported issue, particularly in the pubic bone. However, non-union is frequently asymptomatic and does not require any treatment.

## 6. The PAO Learning Curve

As mentioned above, PAO is considered a rather demanding surgical procedure [36,37]. There have been several articles dealing with the learning curve of this operation [37,38,39]. It is difficult to mention an absolute number as a threshold, but there seems to be some sort of agreement that 30 to 50 PAOs are necessary for complication rates to fall [38,40]. In the first series reported by Ganz et al., clinically relevant complications occurred in the first 18 cases [2]. Davey and Santore reported a significant drop in complication rate from 17% to 2.9% when comparing the first 35 cases to the second 35 cases [38]. The difficulties are frequently attributed to long operating time, blood loss, and trouble in sufficiently mobilizing the fragment to achieve the desired correction. During the learning curve, the risk of not appreciating the structures at risk during every step of the operation may account for the increased likelihood of nerve injury. The majority of published reports are from the first generation of PAO surgeons, who learned the procedure by undertaking fellowship training with Reinhold Ganz in Switzerland [1]. The learning curve with its associated complication is likely the most relevant obstacle in front of the PAO procedure, which limits the number of experts providing the service despite the clinical demand and power of the procedure as a successful treatment.

The coming generations will develop their skills in existing establishments and will bring this technique to new levels. The recent introduction of augmented reality in the orthopedic field will probably find its way into improving technicality aspects of the PAO procedure, easing the technique by accelerating the learning curve and reducing complications [41].

## 7. Bibliometrics around PAO

Now that different aspects of the procedure have been covered, it would be interesting to highlight the bibliometric performance of the science around periacetabular osteotomy. This performance evaluation allows for a reflection of the scholarly impact of the science and literature around PAO [42].

Here, we refer to citation counts as of August 2022. This type of bibliometric analysis has been frequently published. It is no surprise that the most cited article in the field was the article by Reinhold Ganz describing the procedure in 1988 [2]. Interestingly, the second most cited article was by Siebenrock, who reported anteverting PAO for anterior over-coverage.

The ten most published *authors* that have together contributed to more than 500 articles around the PAO procedure since its first description, in descending order, are John Clohisy, Michael Millis, Young Jo Kim, Klaus Siebenrock, Perry L. Schoenecker, Reinhold Ganz, Robert Trousdale, Rafael J. Sierra, Paul E. Beaulé, and Inger Mechlenburg.

The *countries* from which the most articles were published, in descending order, were the USA, Japan, Switzerland, Denmark, and China.

The *institutions* from which most articles were published include the University of Bern Switzerland, Boston Children’s Hospital USA, Washington University USA, Aarhus University Denmark, University of Ottawa Canada, and the University of Fukuoka Japan.

The *journals* that have published the most articles around PAO in descending order include “Clinical Orthopaedics and Related Research”, “The Journal of Bone and Joint Surgery—American Volume”, “The Journal of Hip Preservation Surgery”, “Hip International”, “The American Journal of Sports Medicine”.

## 8. Conclusions

This review encompasses some of the most important pillars of the PAO procedure, starting from its history, and going through its indications, surgical technique, learning curve, and outcome. The literature has many clinical outcome reports by surgeons who have mostly had a direct encounter with the founder of the procedure in Switzerland, from whom they have directly learned. The outcomes are generally very promising, but the learning curve is challenging. Indications have been refined allowing for even more optimized patient selection and outcome improvement. The indications of the procedure have been extended to include acetabular retroversion with posterolateral under-coverage as well as entities of mild or borderline dysplasia.

## 9. Future Directions

A glimpse at the future would reflect major advances related to individual planning, surgical training, and precise surgical conduction. This will include 3D modeling and printing as well as individualized cutting guides that aid the planning and conduction of the procedure [43].

In the era of digitalization, augmented reality will assist in performing bony cuts and act as an aid for some of the blind ischial and retro-acetabular cuts [44]. Innovations in perioperative management will enhance recovery after the procedure and allow for early recovery programs with optimized protocols of pain management [45,46]. Altogether, it seems that the future of this procedure is yet to come. Given that the success of PAO in the young is comparable to the success of hip arthroplasty in the old, PAO should be considered one of the pillars of modern orthopedic surgery.

## Data Availability

Not applicable.
